# Assessment of the Short-Term Effectiveness of Kinesiotaping and Trigger Points Release Used in Functional Disorders of the Masticatory Muscles

**DOI:** 10.1155/2018/5464985

**Published:** 2018-05-10

**Authors:** Danuta Lietz-Kijak, Łukasz Kopacz, Roman Ardan, Marta Grzegocka, Edward Kijak

**Affiliations:** ^1^Independent Unit of Propaedeutic and Dental Physical Diagnostics, Faculty of Medicine and Dentistry, Pomeranian Medical University, Rybacka 1, 70-204 Szczecin, Poland; ^2^Pomeranian Medical University, Rybacka 1, 70-204 Szczecin, Poland; ^3^Department of Econometrics, Faculty of Economic Sciences, Koszalin University of Technology, Kwiatkowskiego 6e, 75-343 Koszalin, Poland; ^4^Scientific Unit of Dysfunction of the Masticatory System, Chair and Department of Prosthodontics, Faculty of Medicine and Dentistry, Pomeranian Medical University, Rybacka 1, 70-204 Szczecin, Poland

## Abstract

Chronic face pain syndrome is a diagnostic and therapeutic problem for many specialists, and this proves the interdisciplinary and complex nature of this ailment. Physiotherapy is of particular importance in the treatment of pain syndrome in the course of temporomandibular joint functional disorders. In patients with long-term dysfunction of masticatory muscles, the palpation examination can localize trigger points, that is, thickening in the form of nodules in the size of rice grains or peas. Latent trigger points located in the muscles can interfere with muscular movement patterns, cause cramps, and reduce muscle strength. Because hidden trigger points can spontaneously activate, they should be found and released to prevent further escalation of the discomfort. Kinesiotaping (KT) is considered as an intervention that can be used to release latent myofascial trigger points. It is a method that involves applying specific tapes to the patient's skin in order to take advantage of the natural self-healing processes of the body. The aim of the study was to evaluate the effect of the kinesiotaping method and trigger points inactivation on the nonpharmacological elimination of pain in patients with temporomandibular disorders. The study was conducted in 60 patients (18 to 35 years old). The subjects were randomly divided into two subgroups of 30 people each. Group KT (15 women and 15 men) were subjected to active kinesiotaping application. Group TrP, composed of 16 women and 14 men, was subjected to physiotherapy with the release of trigger points by the ischemic compression method. The results show that the KT method and TrP inactivation brought significant therapeutic analgesic effects in the course of pain-related functional disorders of the muscles of mastication. The more beneficial outcomes of the therapy were observed after using the KT method, which increased the analgesic effect in dysfunctional patients.

## 1. Introduction

Chronic myofascial pain syndrome is a diagnostic and therapeutic problem for many specialists, such as dentists, laryngologists, neurologists, neurosurgeons, general surgeons, anesthetists, psychiatrists, and oncologists [1]. This indicates the interdisciplinary and complex nature of these diseases. The prevalence of dysfunctional pain syndrome is estimated at around 12% of the adult population and 50% of the elderly population; it is more frequent in women between 20 and 40 years of age [2]. The percentage of women with headache associated with temporomandibular dysfunctions reaches up to 15%, and 10% in men [3]. Pain intensity varies from dull to acute. In people gnashing their teeth at night (occlusal parafunction, bruxism), morning pain in the joint and muscles is characteristic; it intensifies while eating and disappears during the day. In people clenching their teeth during the day, the pain may be most intense in the evening [4]. Signs and symptoms associated with temporomandibular dysfunctions include the pathological wear of the teeth as a result of bruxism, increased muscle tone in the masticatory muscles, overgrowth of the masseter, tinnitus, and changes in the psychological profile [5]. Differential diagnosis of pain in the course of temporomandibular dysfunction should exclude other pathologies of the temporomandibular joint, as well as tumours of the zygomatic bone, neoplasms of the nose, mandible, and parapharyngeal area, systemic connective tissue diseases, giant cell arteritis (mandible claudication), cluster headache, and reflex sympathetic dystrophy of the face [6].

The role of occlusal and nonocclusal parafunctions is emphasized potential etiological factors, along with malocclusion, missing teeth in the lateral areas, macro- and microinjuries of the joint, stress leading to hyperactivity of the masticatory muscles, the activation of masticatory muscles by the route descending from the limbic system and reticular formation, the lack of effective contraction of both lateral pterygoid muscles, and rheumatic diseases [7].

The multiple manifestations of the symptoms lead to a multitude of treatment methods and indicate that there is still no consensus in understanding the pathophysiology of the underlying TMD mechanisms. Because of the heterogeneity of the causes, the treatment of pain syndrome in the course of temporomandibular dysfunction should have a multiprofile character [8, 9].

Temporary pain relief can be obtained by pharmacological treatment with nonsteroidal anti-inflammatory drugs. Drugs that reduce muscle tone, antidepressants, and intra-articular steroids are also used. Dental treatment includes the use of, among others, flexible or hard occlusal splints [10–12]. Physiotherapy is of particular importance in the treatment of pain syndrome in the course of temporomandibular joint functional disorders. Positive therapeutic effects are obtained using botuline, laser therapy, heat therapy, light therapy, electrotherapy, electromagnetic field, manual therapy, proprioceptive neuromuscular motion paving, kinesiotherapy, relaxation techniques, autogenic training, and biofeedback to change of parafunctional behaviors [13, 14].

In patients with long-term dysfunction of masticatory muscles, the palpation examination can localize trigger points, that is, thickening in the form of nodules in the size of rice grains or peas. These are muscle fibres with increased tension, felt as thickening in the course of the muscle fibres. Latent trigger points located in the muscles can interfere with muscular movement patterns, cause cramps, and reduce muscle strength. Because hidden trigger points can spontaneously activate, they should be found and released to prevent further escalation of the discomfort [15]. Physiotherapeutic treatment is primarily based on the inactivation of trigger points by various techniques, such as compressive mobilization, positional release, myofascial relaxation, active relaxation technique, postisometric relaxation technique, or integrated neuromuscular inhibition technique (INIT) [16, 17]. Among the commonly used methods, we should also mention deep tissue massage and passive stretching of the muscles. Kinesiotaping is considered an intervention that can be used to release latent myofascial trigger points. This method involves the use of specific tapes applied to the patient's skin in order to take advantage of the natural self-healing processes of the body. It is very often applied as an element supportive of the therapeutic effect. The action of the method is mainly based on normalizing muscle tension, supporting the work of joints, improving the function of weakened muscles, and increasing microcirculation at the application site [18–20].

## 2. Study Objective

The aim of the study was to evaluate the effect of the kinesiotaping method and trigger points inactivation on the nonpharmacological elimination of pain in patients with temporomandibular disorders.

## 3. Materials and Methods

### 3.1. Material: Inclusion and Exclusion Criteria

The study was conducted during the years 2015-2016 in 60 patients (18 to 35 years old). All qualified patients suffered from painful functional disorders within the masticatory muscles of myofascial characteristic. Patients were also tested by the research diagnostic criteria for temporomandibular disorders (RDC/TMD) introduced by Dworkin and LeResche in 1992 [21]. This enables the standardization of the procedures of epidemiological studies, the unification of TMD diagnostic and exploratory criteria, and the comparison of results of other similar studies. The results of the study were based on the RDC/TMD Axis I diagnostic criteria. All researchers have been trained and calibrated in accordance with the adopted norms presented on the official website of the International RDC/TMD Consortium [22].

The exclusion criteria were as follows: regular drug therapy, mental illness, coagulopathy, diabetes, or chronic infections. The subjects were not addicted to nicotine, alcohol, or drugs. The participants with joint clicking and a clinical diagnosis of disc displacement were also excluded, and they were asked to refrain or not to use self-treatment during the therapy.

The study was approved by the Bioethics Committee of the Pomeranian Medical University in Szczecin (KB–0012/36/15). It is in accordance with ethical standards; all participants signed written informed consent and were acquainted with the technique and the course of the research.

### 3.2. Method

**Figure 1:**
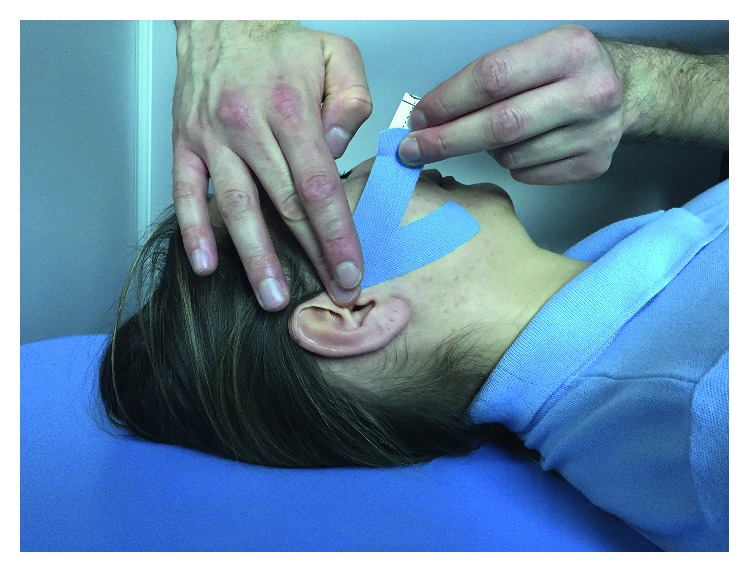
The muscular application of KT to the area of the masseter.

The subjects were randomly divided into two subgroups of 30 people each. Group KT (15 female and 15 male) were subjected to active kinesiotaping application (K-Active Tape Classic, 50 mm × 17 m; Nitto Denko Corporation, Japan) (Figure 1). Subjects undergoing therapy were diagnosed with excessive strain of masseter muscles and muscular pain, without limitations in the movements of the mandible and without disc derangement and joint pain. The muscular application was used for the region of the masseter with a tape (5 cm wide) cut into 2 parts, called tails, which covered the treatment sites without tension. The base was located in the region of the temporomandibular joint. The upper tail ran across the buccal surface of the face towards the nose, while the lower tail was directed towards the chin and thus included the masseter. This type of application raised the surface of the skin, which was translated into a decrease in the tension of the affected area. All participants of the study were obliged to wear the kinesiology tape for a period of 5 days and were advised to carry out everyday activities without unnecessary care.

**Figure 2:**
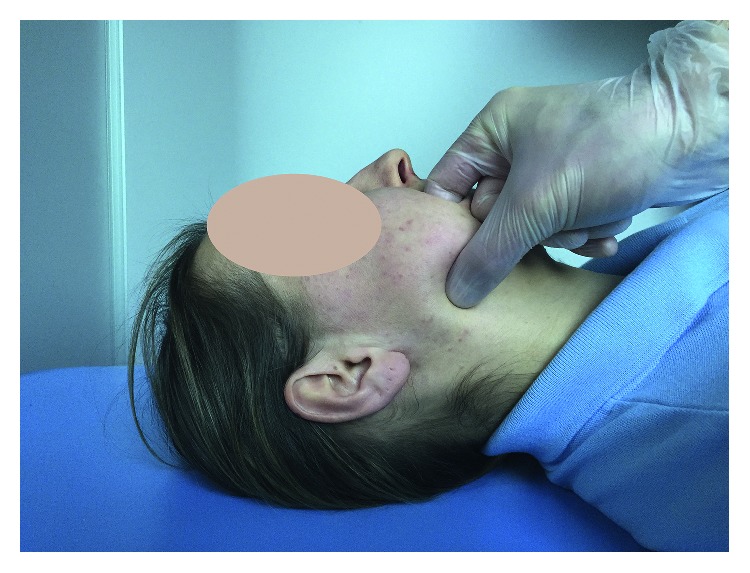
The release of TrP by the ischemic compression method.

Group TrP, composed of 16 women and 14 men, was subjected to physiotherapy with the release of trigger points by the ischemic compression method, which was based on applying pressure to the active trigger point until it was switched off, that is, the pain disappeared (Figure 2). Subjects undergoing therapy were diagnosed with excessive strain of masseter muscles and muscular pain, without limitations in the movements of the mandible and without disc derangement and joint pain. The localization of the trigger points, on average 4 on the right and on the left side, was done palpably with the dental arches clenched, using a pliers grip covering the dense tissue inside and outside the cheek with the thumb and index finger. Trigger point therapy was performed within the upper and lower attachment of the masseter, on the right and left sides. The procedure of deactivation of trigger points was performed three times, on the first, third, and fifth days of therapy.

Before performing the physiotherapeutic procedures in groups KT and TrP and after their application, all patients were subjected to diagnostic actions, including the measurement of pain intensity using the visual analogue scale (VAS). Two dentists were involved in the selection of participants. Only one physiotherapist performed KT applications and TrP therapies. The therapeutic effectiveness of the two methods was verified by comparing the mean values of pain intensity before and after performing the physiotherapeutic procedures. Changes in pain intensity were considered as dependent variable, while the therapy used and patients' gender and age were considered as independent variables.

Prior to the examination, each patient gave written consent to participate in the therapy and was provided with information about the technique and the course of the tests. The protocol was developed in accordance with the latest version of the World Medical Declaration of the Helsinki Association [23]. The patients were also informed about the principle of anonymity of the tests.

## 4. Statistical Analysis

The statistical analysis included determining the physiotherapeutic effects, examining the influence of gender and patient's age on the outcomes, and comparing the analgesic efficacy of the treatment methods. In order to check the efficacy of the therapy, mean pain values were compared before and after performing the procedures. The statistical significance of the change in mean values was verified using the paired sample Welch *t*-test. The linear regression models were also used to examine the influence of gender and age on treatment efficacy.

The hypothesis about greater analgesic efficacy of KT compared to TrP was additionally tested. For this purpose, the mean values of absolute and relative pain intensity changes obtained from two groups of patients were compared using the unpaired sample Welch t-test. (The Welch *t*-test is a generalization of Student's *t*-test for populations with different variances. Significant differences in the value of variance were observed in the measurements taken before and after the treatment, as well as in the comparison of changes after the use of both therapeutic methods.)

## 5. Results

### 5.1. Physiotherapeutic Effects

The basic statistics of patients' age and measurements for the methods applied in particular groups of patients are summarized in Table 1.

The *t*-test confirms the statistical significance of both therapeutic methods in reducing pain symptoms (Table 2.)

### 5.2. Comparison of the Analgesic Effect of KT and TrP

In order to compare the analgesic effect of KT and TrP, absolute change in pain level AbsCh was calculated for each patient as the difference of measurement after and before treatment. In addition, to account for the different levels of initial pain in the two groups of patients entering therapy, relative change RelCh was also calculated:

(1) $$\begin{array}{c} \begin{array}{l} \text{AbsCh}=\text{pain after}-\text{pain before},\\ \text{RelCh}=\frac{\text{AbsCh}}{\text{pain before}}. \end{array} \end{array}$$

Relative change shows what fraction of patient's initial pain was eliminated during treatment.

Although both methods proved to be efficacious, the mean values of the changes after KT and TrP suggest that the KT method gives greater improvement in the reduction of pain. The unpaired sample Welch *t*-test confirms this (Table 3).

### 5.3. Study of the Influence of Gender and Patient's Age on Therapeutic Outcomes

The therapeutic outcomes obtained by using both methods (KT and TrP) in the group of male and female patients were compared by analyzing the mean values of changes using the *t*-test. For both methods, no significant differences were found in the treatment effects between male and female patients.

In the linear regression models, the absolute change in pain intensity was a dependent variable. For both therapy methods, the models with age, gender, and their cross-factor as independent variables were examined. There were no significant variables in any model (*t*-statistic values less than 1).

## 6. Discussion

Face and oral pain syndromes lasting longer than 6 months are a multidisciplinary issue. Close cooperation between neurologists, laryngologist, physiotherapists, psychiatrists, and dentists can help in identifying the causes of these ailments and avoiding the misdiagnosis or false causal relationship. The diagnostic and therapeutic management of facial pain depends on the suspected cause of pain and the accompanying symptoms. It is important to remember that every chronic condition, including pain, causes an impact on the psychological and social status of patients and reduces the quality of life [24].

The study presents cooperation between physiotherapists and dentists by investigating the impact of kinesiotaping and inactivation of trigger points applied to the area of the masseter in the course of functional disorders of the muscles of mastication, with particular emphasis on pain. The original research revealed a significant reduction of pain. The results of the study confirm the observations of other authors who have given special attention to the influence of the kinesiotaping method on the human body.

Youngsook identified changes in myofascial pain and examined the range of movement in the temporomandibular joint after the application of the kinesiotaping method in patients with latent myofascial trigger points within the sternocleidomastoid muscle [25]. He concluded that pain intensity significantly decreased, and the range of motion in the temporomandibular joint considerably increased. Wei-Ting et al. suggest that the KT method can be used as a regular therapy or as a complement to the treatment of myofascial pain [26]. The therapeutic value of the kinesiotaping method in the muscular application is emphasized in the publications of other authors who include this method in the algorithm for treating pain originating from different muscles. Öztürk et al., who used the application of active tapes in patients with myofascial pain syndrome, demonstrated a statistically significant improvement in pain intensity and strength of the upper trapezius muscle [27].

Kalichman et al. described the case of a patient suffering from meralgia paresthetica with symptoms of numbness, paresthesia, and pain in the anterolateral part of the thigh. After using the KT method for 4 weeks, the symptoms significantly regressed and the quality of life improved [28]. Using KT in patients with an acute cervical spine injury, Osterhues showed an immediate decrease in pain intensity in the study group [29].

There are also critical opinions about the use of the kinesiotaping method which we have to take into consideration. Montalvo et al. conducted a meta-analysis of the available literature on KT to assess its efficacy in pain management therapy in patients with musculoskeletal injuries. Articles published between 2003 and 2013 were selected by searching SPORTDiscus, Scopus, ScienceDirect, CINAHL, Cochrane Library, PubMed, and PEDro databases with the terms kinesio tap^∗^, kinesiology tap^∗^, kinesiotap^∗^, and pain. Thirteen articles investigating the effects of kinesiology tape application on pain with at least level II evidence were selected. Combined results of this meta-analysis indicate that KT may have a limited potential to reduce pain in individuals with musculoskeletal injury. The authors suggest using KT in conjunction with or instead of more conventional therapies. However, further research that compares KT to other clinical interventions is needed to evaluate its efficacy [30].

Morris et al. conducted a systematic review of RCTs investigating the use of KT in the management of clinical conditions. A systematic literature search was performed in the following databases: CINAHL, MEDLINE, OVID, AMED, ScienceDirect, PEDro, www.internurse.com, SPORTDiscus, British Nursing Index, www.kinesiotaping.co.uk, www.kinesiotaping.com, Cochrane Central Register of Clinical Trials, and ProQuest.

The review included articles published until April 2012. Evaluation of the risk of bias and the quality of evidence was conducted in accordance with the Cochrane methodology. Eight RCTs met the full inclusion criteria. Six of these included patients with musculoskeletal conditions. There was limited to moderate evidence that KT is no more clinically effective than sham or usual care tape/bandage. There was limited evidence from one moderate quality RCT that KT in conjunction with physiotherapy was clinically beneficial for plantar fasciitis-related pain in the short term. There are, however, serious concerns about the internal validity of this RCT. There is currently insufficient evidence to support the use of KT over other clinical interventions [31].

Parreira et al. conducted a systematic review comparing KT to sham KT. Twelve randomized trials with 495 participants in total were included in the review. The efficacy of KT was assessed in patients with shoulder pain in two trials, knee pain in three trials, chronic low back pain in two trials, neck pain in three trials, plantar fasciitis in one trial, and multiple musculoskeletal conditions in one trial. The methodological quality of the studies that met the inclusion criteria was moderate, with a mean score of 6.1 points on the 10-point PEDro scale. The study found that KT was better than sham KT/placebo and active comparison groups. However, the effect sizes were small and probably not clinically significant or of low quality [32].

In their research, Tremblay and Karam concluded that the application of KT had little effect at the neuromuscular level. The changes in sensory feedback assigned to an elastic tape are probably insufficient to modulate corticospinal excitability in the functional sense [33]. Therefore, more research should be carried out to explain the effectiveness of the KT method in the elimination of TMD-related ailments, which is very difficult due to the need to perform random configurations and double-blind tests. The cosmetic defect caused by sticking active tapes to the skin of the face is also important. Therefore, the analgesic action is local, and its main goal is to enlarge the space between the skin and soft tissues in order to expand the movement space, facilitate the circulation of blood and lymph, and increase the rate of tissue healing, as suggested by Skirven et al. [34].

The pain associated with TMD often reduces the activity of the masticatory muscles. The implementation of the appropriate set of exercises significantly improves the action of analgesic methods and all physical fitness parameters [35]. Because of the multidimensional nature of the problem, patients with musculoskeletal pain of the face should be treated by an interdisciplinary team. There is increasing evidence that psychosocial factors have a significant impact on therapeutic outcomes and may also affect the symptoms reported by patients. Taiminen et al. found that many patients complaining of chronic headache are also diagnosed with a psychiatric or personality disorder that blurs the manifestation of the disease, which considerably affects the treatment [36]. Because a few health care workers believe that they are able to help them on their own and that these people require multidisciplinary treatment, Hals et al. indicate that these patients are often marked as “difficult” [37].

## 7. Conclusions

1. The methods of kinesiotaping (KT) and TrP inactivation have brought significant analgesic effects to the treatment of painful forms of functional disorders of the masticatory muscles.
2. The more beneficial results were observed after using the KT method, which increased the analgesic effect in dysfunctional patients.
3. No influence of gender or patient's age on the treatment results was reported.
4. There is also a need to develop algorithms for the diagnosis and treatment of oral and facial pain syndromes with a strict definition of the role of dentists and physiotherapists.

## Figures and Tables

**Table 1:** The basic statistics of patients' age and pain intensity measurements before and after the application of both methods.

Characteristic	Method of therapy					
	KT			TrP		
	Age (years)	Pain (VAS units)		Age (years)	Pain (VAS units)	
Measurement		Before	After		Before	After
*All patients*						
Minimum	18	3	1	18	4	2
Maximum	35	10	6	35	9	7
Mean	25.87	6.50	3.10	27.37	6.27	4.17
SD	4.86	1.74	1.35	5.08	1.41	1.36
*Male patients*						
Minimum	18	4	1	18	4	3
Maximum	32	8	5	35	9	7
Mean	26.07	5.93	2.93	28.93	6.57	4.50
SD	4.92	1.44	1.10	5.36	1.55	1.30
*Female patients*						
Minimum	19	3	1	19	4	2
Maximum	35	10	6	34	8	6
Mean	25.67	7.06	3.27	26.00	6.00	3.88
SD	4.97	1.87	1.58	4.51	1.25	1.37

KT, kinesiotaping; TrP, trigger point therapy.

**Table 2:** The significance of therapeutic methods' test results.

Method	Pain (VAS units, mean ± SD)		*t*-statistic	*p* value
	Before	After		
KT	6.50 ± 1.74	3.10 ± 1.35	14.92	<0.001
TrP	6.27 ± 1.41	4.17 ± 1.36	8.23	<0.001

**Table 3:** Comparison of the analgesic effect of KT and TrP results.

Characteristic	KT	TrP	*t*-statistic	*p* value
	Mean ± SD	Mean ± SD		
AbsCh	−3.40 ± 1.24	−2.10 ± 1.40	−3.80	<0.001
RelCh	−0.53 ± 0.15	−0.32 ± 0.21	−4.31	<0.001

## Data Availability

All interested in the results and the course of the research are encouraged to contact via e-mail with the Independent Unit of Propaedeutic and Dental Physical Diagnostics, in which the tests were carried out and all data are stored.
